# Macrophage migration inhibitory factor enhances osteoclastogenesis through upregulation of RANKL expression from fibroblast-like synoviocytes in patients with rheumatoid arthritis

**DOI:** 10.1186/ar3279

**Published:** 2011-03-14

**Authors:** Hae-Rim Kim, Kyoung-Woon Kim, Hong Geun Jung, Kwang-Sup Yoon, Hye-Jwa Oh, Mi-La Cho, Sang-Heon Lee

**Affiliations:** 1 Division of Rheumatology, Medical Immunology Center, Department of Internal Medicine, Konkuk University School of Medicine, 1 Hwayang-dong, Kwangjin-gu, Seoul 143-729, Korea; 2 Medical Immunology Center, Institute of Biomedical Science and Technology, Konkuk University, 1 Hwayang-dong, Kwangjin-gu, Seoul 143-729, Korea; 3 Department of Orthopedic Surgery, Konkuk University School of Medicine, 1 Hwayang-dong, Kwangjin-gu, Seoul 143-729, Korea; 4 The Rheumatism Research Center, Catholic Research Institute of Medical Science, The Catholic University of Korea, Seoul, South Korea, 505 Banpo-Dong, Seocho-Ku, Seoul 137-040, Korea

## Abstract

**Introduction:**

Macrophage migration inhibitory factor (MIF) is one of key regulators in acute and chronic immune-inflammatory conditions including rheumatoid arthritis (RA). We examined the effect of MIF on osteoclastogenesis, which is known to play a crucial role in bone destruction in RA.

**Methods:**

The concentration of MIF and receptor activator of nuclear factor-κB ligand (RANKL) in the synovial fluid was measured by ELISA. MIF-induced RANKL expression of RA synovial fibroblasts was determined by real-time PCR and western blot. Osteoclastogenesis was analyzed in culture of human peripheral blood mononuclear cells (PBMC) with MIF. Osteoclastogenesis was also determined after co-cultures of rhMIF-stimulated RA synovial fibroblasts with human PBMC.

**Results:**

Synovial fluid MIF concentration in RA patients was significantly higher than in osteoarthritis (OA) patients. The concentration of RANKL correlated with that of MIF in RA synovial fluids (*r *= 0.6, *P *< 0.001). MIF stimulated the expression of RANKL mRNA and protein in RA synovial fibroblasts, which was partially reduced by blocking of interleukin (IL)-1β. Osteoclasts were differentiated from PBMC cultures with MIF and M-CSF, even without RANKL. Osteoclastogenesis was increased after co-culture of MIF-stimulated RA synovial fibroblasts with PBMC and this effect was diminished by RANKL neutralization. Blocking of PI3 kinase, p38 MAP kinase, JAK-2, NF-κB, and AP-1 also led to a marked reduction in RANKL expression and osteoclastogenesis.

**Conclusions:**

The interactions among MIF, synovial fibroblasts, osteoclasts, RANKL, and IL-1β have a close connection in osteoclastogenesis and they could be a potential gateway leading to new therapeutic approaches in treating bone destruction in RA.

## Introduction

Macrophage migration inhibitory factor (MIF) plays a crucial role in rheumatoid arthritis (RA) pathogenesis, linking the innate and adaptive immune responses [1,2]. As well as its role in inflammatory responses, MIF takes part in the destructive process in RA. In RA joint destruction, matrix metalloproteinases (MMP) are thought to play an important role in synovial invasion [3,4]. Various MMPs are upregulated in RA synovial fluid and synovium [4-6], and MIF upregulates MMP-1, MMP-2, and MMP-3 expression in RA synovial fibroblasts [4,6]. MIF also induces MMP-9 and MMP-13 in rat osteoblasts [7]. Besides the induction of MMPs, MIF participates indirectly in joint destruction by promoting angiogenesis in RA synovial fibroblasts [8] and inducing many osteoclast (OC)-inducing molecules such as TNF-α, IL-1, IL-6, and prostaglandin E_2 _(PGE_2_) [1,2,9,10].

MIF-deficient mice are resistant to ovariectomy-induced bone loss and MIF transgenic mice have high-turnover osteoporosis, suggesting that MIF could mediate bone resorption during bone remodeling and balance [11,12]. MIF also upregulates the expression of receptor activator of nuclear factor-κB ligand (RANKL) mRNA in murine osteoblasts. MIF has no effect on bone formation_, _indicating that it might play a role in the physiological or pathological metabolism of bone, especially in bone resorption [12]. However, a recent study suggests that MIF inhibits osteoclastogenesis, based on the result that MIF inhibits OC formation in murine bone marrow (BM) cultures in the presence of RANKL. BM cells from MIF knockout mice had an increased capacity to form OC, and MIF knockout mice had decreased trabecular bone volume with low turnover [13].

To date, the effects of MIF on osteoclastogenesis have not been studied in the context of human disease systems. Two clinical studies suggest that MIF might be involved in joint destruction in RA patients. Greater circulating MIF levels correlate with more severe radiographic joint damage [14], and the MIF concentration of synovial fluid is significantly higher in RA patients with bony erosion than in those without [8]. RA joint destruction is closely related to osteoclastogenesis and the major inducer of OC, RANKL. So, we hypothesized that MIF may play an important role in the process of bone destruction in RA patients through the induction of RANKL or direct involvement of osteoclastogenesis. Thus we needed a greater understanding of the relation between MIF and the pathogenesis of bony destruction in RA. In this study, we determined the effect of MIF on RANKL induction in human RA synovial fibroblasts, the relation of RANKL and MIF, and the role of MIF in OC differentiation in RA patients.

## Materials and methods

### Patients

Synovial fluids were obtained from 16 RA patients fulfilling the 1987 revised criteria of the American College of Rheumatology (formerly the American Rheumatism Association) [15]. Informed consent was obtained from all patients, and the experimental protocol was approved by the Institutional Review Board for Human Research, Konkuk University Hospital (KUH1010186). Synovial tissues were isolated from eight RA patients (mean age 63.4 ± 4.6, range 38 to 76 years) undergoing total knee replacement surgery.

### Isolation of synovial fibroblasts

Synovial fibroblasts were isolated by enzymatic digestion of synovial tissues obtained from RA patients undergoing total joint replacement surgery, as described previously [16].

### Reagents

Recombinant human (rh) MIF, rhRANKL and rh monocyte-colony stimulating factor (M-CSF) were purchased from R&D Systems (Minneapolis, MN, USA). Parthenolide, curcurmin and cyclosporin A were obtained from Sigma Chemical Co. (St. Louis, MO, USA). LY294002, SB203580, SP600125, PD98059, and AG490 were obtained from Calbiochem (Schwalbach, Germany). Anti-human IL-1β, TNF-α, IL-6, RANKL and MIF were purchased from R&D Systems (Minneapolis, MN, USA).

### Determination of concentrations of soluble RANKL and MIF by sandwich ELISA

Concentrations of soluble (s) RANKL and MIF in sera and synovial fluids were measured by sandwich ELISA as described previously [16].

### Immunohistochemistry of RA synovium and synovial fibroblasts

Immunohistochemical staining for RANKL and MIF was performed on sections of synovium. Briefly, synovium samples were obtained from patients, fixed in 4% paraformaldehyde solution overnight at 4°C, dehydrated with alcohol, washed, embedded in paraffin, and sectioned into slices 7 μm thick. The sections were depleted of endogenous peroxidase activity by adding methanolic H_2_O_2 _and were blocked with normal serum for 30 minutes. After overnight incubation at 4°C with polyclonal anti-human RANKL and anti-MIF antibodies (Santa Cruz Biotechnology, Santa Cruz, CA, USA), the samples were incubated with the appropriate secondary antibodies biotinylated anti-rabbit IgG or biotinylated anti-goat IgG for 20 minutes and then incubated with streptavidin-peroxidase (Vector, Peterborough, UK) for one hour followed by incubation with 3,3"-diaminobenzidine (Dako, Glostrup, Denmark) for five minutes. The sections were counterstained with hematoxylin. Samples were photographed with an Olympus photomicroscope (Tokyo, Japan). Synovial fibroblasts were grown in 150 mm dishes in DMEM complete medium, plated at a density of 1 × 10^4 ^cells/cm^2 ^onto glass coverslips (12 mm diameter), and stimulated with rhMIF (0.1, 1, 5, and 10 ng/mL) (R&D Systems, Minneapolis, MN, USA). Cells were fixed in 4% paraformaldehyde for immunohistochemical analysis using anti-RANKL antibody 72 hours after the addition of rhMIF.

### Expression of RANKL mRNA measured by real-time reverse transcription polymerase chain reaction amplification

RA synovial fibroblasts were stimulated with rhMIF (0.1, 1, 5, and 10 ng/mL). For signal pathway analysis of RANKL, synovial fibroblasts were incubated in the presence or absence of LY294002 (20 μM), SB203580 (10 μM), SP600125 (1 μM), PD98059 (10 μM), AG490 (50 μM), cyclosporin A (100 nM), parthenolide (10 μM), or curcumin (10 μM) for one hour before the addition of rhMIF. After incubation for 72 hours, mRNA was extracted using RNAzol B (Biotex Laboratories, Houston, TX, USA) according to the manufacturer's instructions. RT-PCR of 2 μg of total mRNA was carried out at 42°C using the SuperScript™ reverse transcription system (Takara, Shiga, Japan). PCR was performed in a 20 μl final volume in capillary tubes in a LightCycler instrument (Roche Diagnostics, Mannheim, Germany). The reaction mixture contained 2 μl of LightCycler FastStart DNA MasterMix for SYBR^® ^Green I (Roche Diagnostics, Mannheim, Germany), 0.5 μM of each primer, 4 mM MgCl_2_, and 2 μl of template DNA. All capillaries were sealed, centrifuged at 500 *g *for five seconds and then amplified in a LightCycler instrument, with activation of polymerase (95°C for 10 minutes), followed by 45 cycles of 10 seconds at 95°C, 10 seconds at 60°C (for β-actin control) and at 59°C (RANKL), and 10 seconds at 72°C. The temperature transition rate was 20°C/second for all steps. The double-stranded PCR product was measured during the 72°C extension step by detection of fluorescence associated with the binding of SYBR Green I to the product. Fluorescence curves were analyzed with LightCycler software (v. 3.0; Roche Diagnostics, Mannheim, Germany). The relative expression level of each sample was calculated as the level of RANKL, tartrate-resistant acid phosphatase (TRAP), cathepsin K, calcitonin receptor, or MMP-9 normalized to the endogenously expressed housekeeping gene for β-actin. Melting curve analysis was performed immediately after the amplification protocol under the following conditions: 0 seconds (hold time) at 95°C, 15 seconds at 71°C, and 0 seconds (hold time) at 95°C. The rate of temperature change was 20°C/second, except for 0.1°C/second in the final step. The melting peak generated represented the amount of specific amplified product. The crossing point (*C*_p_) was defined as the maximum of the second derivative from the fluorescence curve. Negative controls were included and contained all elements of the reaction mixture except template DNA. All samples were processed in duplicate.

### Western blot analysis

Synovial fibroblasts were incubated with rhMIF for 30 minutes, a whole cell lysate was prepared from about 2 × 10^5 ^cells by homogenization in the lysis buffer, and the lysate was centrifuged at 14,000 rpm for 15 minutes. The protein concentration in the supernatant was determined using the Bradford method (BioRad, Hercules, CA, USA). Protein samples were separated on 10% SDS-PAGE and transferred to a nitrocellulose membrane (Amersham Pharmacia Biotech, Uppsala, Sweden). For western hybridization, the membrane was preincubated with 0.5% skim milk in Tris-buffered saline (TBS) with 0.1% Tween 20 (TTBS) at room temperature for two hours. The primary antibody to phospho-Akt, phospho-STAT3, phospho-IκBα, phospho-c-Jun (Cell Signaling Technology Inc, Danvers, MA, USA) or phospho-p38 mitogen-activated protein kinase (MAPK; Santa Cruz Biotechnology Inc., Santa Cruz, CA, USA) diluted 1:1000 in 5% bovine serum albumin, TTBS, was added and incubated overnight at 4°C. The membrane was washed four times with TTBS, horseradish peroxidase-conjugated secondary antibody was added, and the membrane was incubated for one hour at room temperature. After TTBS washing, the hybridized bands were detected using an ECL detection kit and Hyperfilm-ECL reagents (Amersham Pharmacia Biotech, Uppsala, Sweden).

### Monocyte isolation

Peripheral blood mononuclear cells (PBMC) were separated by Ficoll-Hypaque (Sigma Chemicals, Poole, Dorset, UK) density gradient centrifugation from buffy coats obtained from healthy volunteers. The cells were washed three times with sterile phosphate-buffered saline (PBS) and resuspended in RPMI 1640 (Life Technologies, Grand Island, NY, USA) supplemented with 10% fetal bovine serum (FBS), 2 mM l-glutamine, and 1% penicillin/streptomycin, henceforth called complete medium. Freshly isolated PBMCs were incubated at 37°C in complete medium and allowed to adhere for 45 minutes. The nonadherent cells were removed and the adherent cells were washed with sterile PBS, harvested with a rubber policeman, and stained with monocyte-specific anti-CD14 monoclonal antibody to assess the purity of the preparation. Of the isolated cells, 90% expressed CD14.

### Osteoclast formation

RA synovial fibroblasts were seeded into 12-well multiwell dishes (5 × 10^3 ^cells/well) and stimulated with rhMIF for three days. As described above, isolated human monocytes (5 × 10^4 ^cells/well) were added to the stimulated fibroblasts with fresh media. The cells were cocultured for three weeks in α-minimal essential medium (MEM) and 10% heat-inactivated FBS in the presence of 25 ng/mL of rhM-CSF. The medium was changed on day three and then every other day. The addition of rhRANKL protein, prepared as described previously [17], was used as a positive control. On day 21, TRAP-positive cells were identified using a leukocyte acid phosphatase kit according to the manufacturer's recommended protocol (Sigma-Aldrich, Poole, Dorset, UK) [18].

### Statistical analysis

Data are expressed as the mean ± standard deviation (SD). Statistical analysis was performed using the Mann-Whitney *U *test for independent samples and the Wilcoxon signed rank test for related samples. *P *values less than 0.05 were considered significant.

## Results

### The relation between soluble RANKL and MIF in synovial fluid of RA patients

The clinical characteristics of the 16 RA patients were as follows: age 49.4 ± 2.5 years, disease duration 82.2 ± 12.4 months, erythrocyte sedimentation rate 42.7 ± 6.2 mm/h, and C-reactive protein 1.69 ± 0.3 mg/dL.

**Figure 1:**
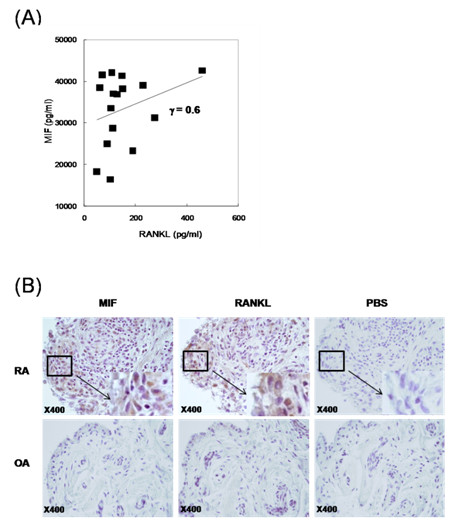
**The expression of MIF and RANKL in RA human synovial fluid and synovium**. **(a) **Correlation between the levels of MIF and sRANKL in synovial fluids from 16 patients with RA. **(b) **Immunohistochemical detection of MIF and RANKL in the synovium of patients with RA and OA. All tissues were counterstained with hematoxylin (original magnification 400×). MIF, macrophage migration inhibitory factor; OA, osteoarthritis; RA, rheumatoid arthritis; RANKL, receptor activator of nuclear factor kappa-B ligand.

To determine the relation of MIF with sRANKL, the concentrations of sRANKL and MIF in synovial fluid from RA patients were measured using sandwich ELISA. In RA patients, the synovial sRANKL concentration correlated with the synovial MIF concentration in RA patients (γ = 0.6; *P *< 0.001; Figure 1a), but the serum sRANKL concentration did not correlate with serum MIF concentration (γ = 0.13; *P *= 0.5, data not shown). We used immunohistochemical staining to compare the expression of MIF and RANKL in synovial tissues. More intense staining of MIF and RANKL was observed in synovium from patients with RA compared with synovium from patients with osteoarthritis (OA). RANKL expression consistently overlapped with that of MIF (Figure 1b).

### MIF induces RANKL expression mediated by IL-1β in RA human synovial fibroblasts

**Figure 2:**
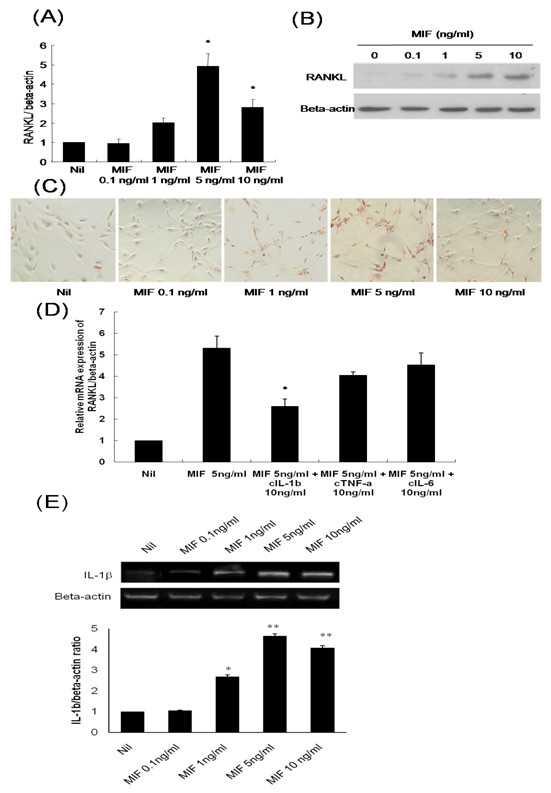
**The effect of MIF on the expression of RANKL in RA human synovial fibroblasts**. **(a) **Isolated RA synovial fibroblasts were incubated with rhMIF (0.1 to 10 ng/mL) for 72 hours, and mRNA was extracted and measured using real-time PCR. **(b) **Isolated RA synovial fibroblasts were incubated with rhMIF (0.1 to 10 ng/mL) for 72 hours, and protein was extracted and measured using western blot analysis. **(c) **RA synovial fibroblasts were cultured with 0.1 to 10 ng/mL of rhMIF for 72 hours and stained with an anti-RANKL antibody (red) (original magnification 400×). **(d) **Effect of neutralizing agents for known osteoclastogenic factors on MIF-induced RANKL expression. RA synovial fibroblasts were treated with rhMIF 5 ng/mL for 72 hours in the presence or absence of anti-IL-1β, anti-TNF-α, or anti-IL-6. RANKL mRNA expression was quantified using real-time PCR. **(e) **Isolated RA synovial fibroblasts were incubated with rhMIF (0.1 to 10 ng/mL) for 72 hours, and MIF-induced IL-1β mRNA expression was measured using RT-PCR. The data represent the mean and standard deviation of three separate experiments. *P < 0.05 and **P < 0.005. MIF, macrophage migration inhibitory factor; RA, rheumatoid arthritis; RANKL, receptor activator of nuclear factor kappa-B ligand.

After RA synovial fibroblasts were stimulated with rhMIF, the expression of RANKL mRNA and protein was determined using real-time PCR, western blot, and intracellular immunostaining. The expression of RANKL mRNA and protein was increased in a dose-dependent manner by rhMIF stimulation. The expression of RANKL mRNA was maximal after stimulation with 5 ng/mL rhMIF at 72 hours (Figure 2a). There was a little difference in the expression of RANKL protein which was maximal with 10 ng/mL of rhMIF (Figure 2b). RANKL expression also increased in the cultured RA synovial fibroblasts, as shown by *in vitro *cellular immunostaining 72 hours after MIF stimulation, with a similar dose response to that demonstrated by real-time PCR (Figure 2c). There was neither a cytotoxic effect nor a proliferative effect on RA synovial fibroblasts at the experimental doses of rhMIF (data not shown).

There was no additive effect on RANKL expression after stimulation with combinations of rhMIF and other cytokines such as TNF-α, IL-1β, and stromal cell-derived factor (SDF)-1 (data not shown). After blocking IL-1β, MIF-induced RANKL expression was partially decreased, but the blockage of TNF-α or IL-6 had no influence on MIF-induced RANKL expression (Figure 2d). As MIF-induced RANKL expression was decreased after IL-1β inhibition, we examined the effect of MIF on IL-1β expression in RA synovial fibroblasts. MIF also stimulated IL-1β mRNA expression and the effect was also maximal in a dose of 5 ng/ml at 72 hours (Figure 2e).

### Intracellular signals involved in MIF-induced RANKL expression in RA human synovial fibroblasts

**Figure 3:**
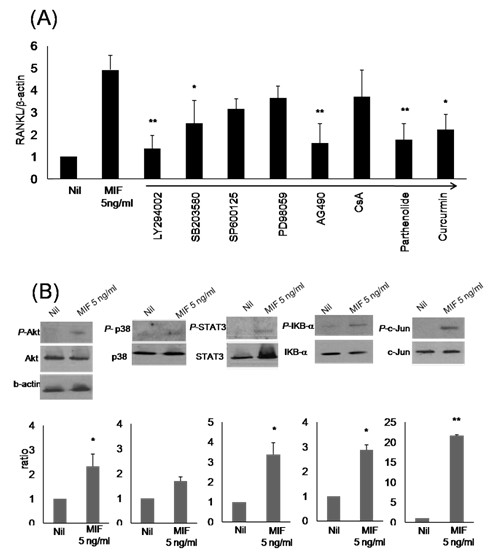
**Effect of signal inhibitors on MIF-induced RANKL expression in RA human synovial fibroblasts**. RA synovial fibroblasts were pretreated with 20 μM LY294002, 10 μM SB203580, 1 μM SP600125, 10 μM PD98059, 50 μM AG490, 100 nM cyclosporin A, 10 μM parthenolide, or 10 μM of curcurmin, then cultured with 5 ng/mL of rhMIF for 72 hours. **(a) **After RA synovial fibroblasts were incubated with the signal inhibitors and rhMIF, mRNA was extracted and measured using real-time PCR. The data represent the mean and standard deviation of five separate experiments. **(b) **MIF activates the phosphorylation of Akt, p38 MAPK, STAT3, IκBα, and c-Jun in RA synovial fibroblasts. The activated forms of Akt, p38 MAPK, STAT3, IκBα, and c-Jun were detected by western blot analysis in RA synovial fibroblasts stimulated with rhMIF, while the amounts of total Akt, p38 MAPK, STAT3, IκBα, and c-Jun were unchanged. The data represent one of three independent experiments. *P < 0.05 and **P < 0.005. MIF, macrophage migration inhibitory factor; RA, rheumatoid arthritis; RANKL, receptor activator of nuclear factor kappa-B ligand.

To determine the signal transduction pathways mediating the MIF induction of RANKL expression, we used 20 μM LY294002 as a phosphatidylinositol (PI)-3 kinase inhibitor, 10 μM SB203580 as a p38 MAPK inhibitor, 1 μM SP600125 as a c-Jun N-terminal kinase (JNK) inhibitor, 10 μM PD98059 as a MAP kinase kinase-1 (MEK1) inhibitor, 50 μM AG490 as a Janus kinase 2 (JAK-2) inhibitor, 100 nM cyclosporin A as a calcineurin inhibitor, 10 μM parthenolide as a NF-κB inhibitor, and 10 μM curcurmin as an activator protein (AP)-1 antagonist. RA synovial fibroblasts were preincubated for one hour in the presence of the different signal inhibitors, and then stimulated using 5 ng/mL of rhMIF for 72 hours for PCR and 30 minutes for western blot, respectively. The expression of RANKL mRNA was determined by real-time PCR. The expression of RANKL mRNA was completely blocked after inhibiting the activities of PI3K, STAT3, and NF-κB (*P *< 0.005). The expression of RANKL mRNA was also partially blocked after inhibition of p38 MAPK and AP-1 (*P *< 0.05). In contrast, the inhibition of JNK, ERK, and calcineurin activities had no effect on MIF-induced RANKL expression (Figure 3a). Cytotoxic effects on synovial fibroblasts of the chemical inhibitors at experimental concentrations were not observed (data not shown). MIF activates the phosphorylation of Akt, p38 MAPK, STAT3, IκBα, and c-Jun in RA synovial fibroblasts. The activated forms of Akt, p38 MAPK, STAT3, IκBα, and c-Jun were detected by western blot analysis in RA synovial fibroblasts stimulated with rhMIF. The ratio of phosphorylated molecules and total proteins was calculated as 2.33, 1.69, 3.4, 2.88, and 21.6, respectively (Figure 3b).

### MIF induces osteoclastogenesis through the upregulation of RANKL expression by RA human synovial fibroblasts

**Figure 4:**
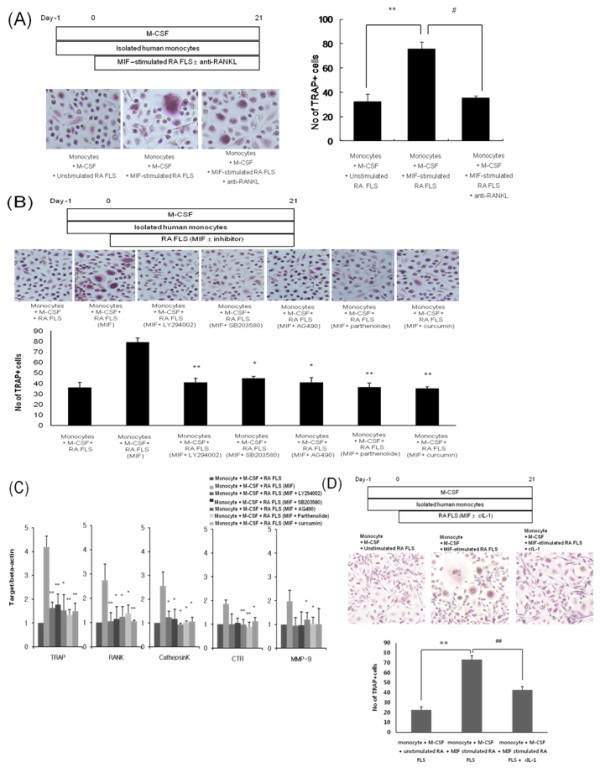
**Cocultures of monocytes and MIF-activated RA human synovial fibroblasts showed significantly more TRAP-positive multinucleated cells**. **(a) **Schematics for cocultures of isolated human monocytes and RA synovial fibroblasts prestimulated with rhMIF for OC differentiation. RA synovial fibroblasts were pretreated with rhMIF 5 ng/mL for three days, monocytes were added to each well, and cocultures were maintained in α-MEM containing 10% horse serum and 25 ng/mL M-CSF for 21 days. TRAP-positive multinucleated cells were counted. Results are presented as the mean and standard deviation (SD) of four separate experiments. #*P *< 0.05 and **P < 0.005. **(b) **Schematics of cocultures of isolated human monocytes and RA synovial fibroblasts prestimulated by rhMIF with or without signal inhibitors for OC differentiation. RA synovial fibroblasts were pretreated with rhMIF 5 ng/mL for three days, monocytes were added to each well with the different signal inhibitors, and cocultures were maintained in α-MEM containing 10% horse serum and 25 ng/mL M-CSF for 21 days. TRAP-positive multinucleated cells were counted. Data represent the mean and SD of four separate experiments. *P < 0.05 and **P < 0.005. **(c) **The expression of TRAP, RANK, cathepsin K, CTR, MMP-9, and β-actin mRNA from differentiated OCs that were cocultured with MIF-stimulated RA synovial fibroblasts was measured using quantitative real-time PCR. **(d) **Differentiation into TRAP-positive multinucleated cells was decreased after blocking of IL-1β. **P < 0.005 versus monocyte + M-CSF + unstimulated RA FLS and *P *< 0.005 versus monocyte + M-CSF + MIF stimulated RA FLS. The data represent the mean and SD of four separate experiments. CTR, calcitonin receptor; FLS, fibroblast-like synoviocytes; MIF, macrophage migration inhibitory factor; MMP, matrix metalloproteinases; OC, osteoclast; RA, rheumatoid arthritis; TRAP, tartrate resistant acid phosphatase.

PBMC can differentiate into TRAP-positive multinucleated OCs in the presence of RANKL and M-CSF [19-21]. Isolated human PBMC were cocultured with MIF-prestimulated RA synovial fibroblasts in the presence of M-CSF, and then TRAP-positive multinucleated cells were also differentiated (*P *< 0.005). When the monocytes were cocultured with MIF-prestimulated RA synovial fibroblasts in the presence of anti-RANKL antibodies, OC formation was significantly decreased (*P *< 0.05, Figure 4a). Next, isolated PBMC were cultured with MIF-stimulated RA synovial fibroblasts and different signal inhibitors. After signal inhibition using LY294002, SB203580, AG490 (*P *< 0.05), parthenolide (*P *< 0.005), and curcurmin differentiation into OCs was significantly decreased (Figure 4b). The expression of other osteoclastogenic markers, such as RANK, cathepsin K, calcitonin receptor (CTR) and MMP-9 was also determined by real-time PCR. Their relative mRNA expression correlated well with the counted number of TRAP positive OCs (Figure 4c). Meanwhile, after monocytes and MIF-prestimulated RA synovial fibroblasts were cultured in the presence of anti-IL-1 antibody, the differentiation of TRAP-positive OC was decreased (Figure 4d).

### MIF induced osteoclastogenesis in human monocytes via PI3K, p38 MAPK, NF-κB, and AP-1 pathways

**Figure 5:**
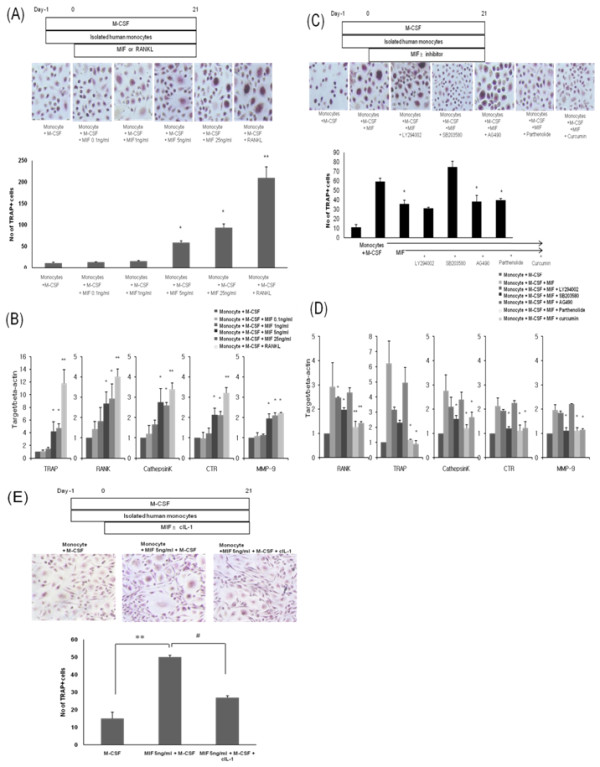
**Human OC differentiation induced by MIF**. **(a) **Schematics for OC differentiation after isolated human PBMC were cultured with 25 ng/mL of M-CSF and 100 ng/mL of rhRANKL or 0.1, 1, 5, 25 ng/mL of rhMIF. TRAP-positive multinucleated cells were counted. Results are presented as the mean and standard deviation (SD) of four separate experiments. **(b) **The expression of TRAP, RANK, cathepsin K, CTR, MMP-9 by the differentiated OCs was measured using quantitative real-time PCR. Data represent the mean and SD of four separate experiments. **(c) **Schematics for OC differentiation after isolated human monocytes were cultured with 25 ng/mL M-CSF and 5 ng/mL rhMIF with 20 μM LY294002, 10 μM SB203580, 50 μM AG490, 10 μM parthenolide, or 10 μM curcurmin. TRAP-positive multinucleated cells were counted. Data represent the mean and SD of four separate experiments. **(d) **The expression of TRAP, RANK, cathepsin K, CTR, MMP-9 by the differentiated OCs that were cultured with rhMIF was measured using quantitative real-time PCR. *P < 0.05 and **P < 0.005. **(e) **Differentiation into TRAP-positive multinucleated cells was decreased after blocking of IL-1β. **P < 0.005 versus M-CSF and ^**#**^*P *< 0.05 versus MIF 5ng/ml + M-CSF. Data represent the mean and SD of four separate experiments. CTR, calcitonin receptor; MIF, macrophage migration inhibitory factor; MMP, matrix metalloproteinases; OC, osteoclast; PBMC, peripheral blood mononuclear cell; RA, rheumatoid arthritis; RANKL, Receptor activator of nuclear factor kappa-B ligand; TRAP, Tartrate resistant acid phosphatase.

To evaluate whether MIF, like RANKL, has a role in the differentiation of monocytes into OCs, human PBMC were cultured with rhMIF and rhM-CSF. Under the influence of MIF and M-CSF, TRAP-positive multinucleated cells, such as OCs, differentiated from the monocytes, but the effect of MIF seemed to be less than the effect of RANKL (*P *< 0.05). When the monocytes were cultured with various doses of rhMIF, OC formation was significantly increased in a dose-dependent manner (Figure 5a). The expression of OC surface markers was also increased after treatment with rhMIF (Figure 5b).

To analyze the intracellular signal pathways mediating MIF-induced OC differentiation, the isolated monocytes were cultured with rhMIF and different signal inhibitors. The adhesion of the monocytes and their differentiation into TRAP-positive OCs was significantly decreased after culture with LY294002, parthenolide, curcurmin (*P *< 0.05), and SB203580 (*P *< 0.005; Figure 5c). The expression of OC surface markers was also decreased after treatment of LY294002, SB203580, parthenolide, and curcumin (*P *< 0.05; Figure 5d).

After inhibition of IL-1β, differentiation into OC was decreased and the size of nucleus was also decreased (Figure 5e).

## Discussion

Synovitis and bony destruction are pathophysiological characteristics of RA, and marginal bony erosion, periarticular osteopenia, and joint space narrowing are the radiographic hallmarks of RA [22,23]. Synovial inflammation and bony destruction are closely related processes [24], but contrary to synovitis, the bony changes are usually irreversible and accumulate with time, and can bring about joint dysfunction and an unfavorable disease outcome [25,26]. As a result, RA causes significant socioeconomic impact because of physically disabled and unemployed people [27,28].

Both cellular mechanisms and various inflammatory mediators are involved in the pathogenesis of bone erosion in RA, forming complex networks [24,29]. Among these process, OCs are the essential cells involved in the cellular mechanisms of the process of bony erosion [30-32]. In RA synovium, OCs are found at the pannus-bone and pannus-subchondral bone junctions of arthritic joints, forming erosive pits in the bone [30,31]. Two additional cells play important roles in osteoclastogenesis: synovial fibroblasts and activated T cells. They express RANKL in the inflamed synovium, which promotes osteoclastogenesis, and also express cathepsin K at sites of synovial bone destruction [33]. RANKL is the key molecule in OC differentiation and the augmentation of activity and survival of these cells, and is often called OC differentiation factor (ODF). In the serum transfer model of arthritis in the RANKL knockout mouse, the synovial inflammation and cartilage erosions are similar to those in wild-type mice, but the degree of bony erosion is significantly reduced [34]. This result confirms the essential role of RANKL in the pathogenesis of bone erosion, regardless of inflammation or cartilage damage. The expression of RANKL is regulated by proinflammatory mediators such as TNF-α, IL-1, IL-6, IL-17, and PGE_2 _[35]. These inflammatory molecules are abundant in RA synovium, so the inflamed synovium supplies an optimal environment for RANKL activation.

In this study, we determined the relation between bony erosion and MIF in human RA. In the previous studies, MIF induces TNF-α, IL-1, IL-6, and PGE_2_, which in turn promote RANKL expression [1,2,9,10,36], and the synovial MIF concentration is higher in RA patients with bony erosion than in those without [8]. Based on these results, we hypothesized that MIF might have a role in the pathogenesis of bone erosion, that is, it could have a direct effect on OC differentiation and an indirect effect on the induction of other inflammatory mediators that induce RANKL expression. First, we measured the synovial concentrations of MIF and RANKL in RA patients. Synovial fluid MIF concentration was higher in RA patients than in controls, as in our previous study [8], but the synovial RANKL concentration did not differ between RA patients and controls. In previous studies, serum and synovial RANKL levels were higher in RA patients than in controls [37], but the RANKL level was not related to any measures for disease activity [38]. In contrast, we found that the serum and synovial MIF concentration was well correlated with RA disease activity [8,14]. Compared with previous studies, the patients enrolled in this study had longer disease duration and less active disease [37], so MIF may reflect disease activity more closely than does RANKL. In this study, synovial RANKL concentration was significantly correlated with synovial MIF concentration, and this observation led us to investigate their close relation in the RA synovial tissues.

We investigated the effect of MIF on RANKL expression in RA synovial fibroblasts. Synovial fibroblasts, such as activated T cells, are major sources of the RANKL that promotes OC differentiation and bone erosion [33]. Like other proinflammatory cytokines, MIF stimulates the expression of RANKL mRNA and protein in RA synovial fibroblasts, but there was no additive effect with other proinflammatory cytokines such as TNF-α and IL-1β. After blocking IL-1β, MIF-induced RANKL expression was partially decreased. This result suggests that RANKL expression was directly induced by MIF and also that it was indirectly stimulated by MIF-induced IL-1β. IL-1β has the potential to induce OC differentiation and RANKL expression, and overexpressed MIF could induce some inflammatory mediators, such as IL-1β in RA synovium, resulting in upregulation of RANKL and promotion of OC differentiation. Therefore, the MIF-IL-1β-RANKL interaction could be a major axis involved in RA bone erosion.

We investigated the effect of MIF on OC differentiation. We substituted MIF for RANKL in the traditional culture system for OC differentiation. After isolated PBMC were cultured with rhMIF and M-CSF, the numbers of TRAP-positive multinucleated cells were counted. OC developed in this new system without RANKL, but the degree of OC differentiation by MIF was less than that of RANKL. This result showed that MIF is one of the inflammatory cytokines involved in osteoclastogenesis, even if RANKL is the major molecule that induces OC differentiation. We also demonstrated that MIF-prestimulated RA synovial fibroblasts have a potential effect on osteoclastogenesis when the cells are co-cultured with PBMC. This culture system is more practical in an *in vitro *system similar to human RA synovium. RA synovial fibroblasts are exposed to a variety of cytokines that promote inflammation, and when these ailing cells encounter OC precursors, they could induce osteoclastogenesis by cytokine production or direct interaction between cells. This study was focused on the indirect osteoclastogenic effect mediated by RA synovial fibroblasts and RANKL, but MIF could directly enhance osteoclastogenesis from monocytes in the absence of additional RANKL. These two pathways imply more distinct and reinforced mechanisms for MIF-induced osteoclastogenesis, and a tipping point such as MIF production could be a potential therapeutic target.

In contrast to our results, a recent study suggests that MIF inhibits osteoclastogenesis [13]. Although MIF enhances the expression of RANKL mRNA in murine osteoblasts and the expression of RANKL mRNA is enhanced in MIF transgenic mice, MIF inhibits OC formation in bone marrow cultures by decreasing fusion and decreasing the number of nuclei. The number of TRAP-positive OC is greater in MIF-deficient mice than in wild type mice, and the addition of MIF to the cells decreased TRAP-positive OC formation. Therefore, it appears that MIF plays an inhibitory role in bone resorption. The discrepancy between two studies could be explained by several differences in study systems. First, our study used human PBMC, whereas the former study used osteoclast precursor cells from MIF knockout mice. MIF inhibits osteoclast formation *in vitro *in wild type mice bone marrow cell cultures and in the RAW264.7 macrophage cell line. Based on these data, MIF appears to directly inhibit osteoclastogenesis *in vitro *but its effects on osteoclasts *in vivo *are complex and may result from decreased RANKL expression in the osteoclast precursor cells from MIF knockout mice that were exposed to low levels of RANKL *in vivo *and as a result these cells have increased sensitivity to RANKL *in vitro *when cultured at high density.The MIF knockout mice that they used, had a marked resistance to lipopolysaccharide-induced endotoxic shock, and decreased TNFα production in response to lipopolysaccharide treatment. TNF-a also acts directly on the osteoclast precursor to potentiate RANKL-induced osteoclastogenesis, even in the absence of elevated levels of RANKL. MIF knockout mice were used in the previous paper, and had inhibited TNF production. Thus, osteoclast formation may have been inhibited. Second, we put the focus on an actual inflammatory disease of humans. In human RA synovial fibroblasts, the over-expressed MIF induces other inflammatory mediators, and then the inflammatory mediators, such as RANKL and IL-1β, enhance and potentiate osteoclastogenesis. Third, the former study treated RANKL with MIF in the OC differentiation system, but we did not treat RANKL in the culture system. More intensive study will be needed for explaining these conflicting results. We hypothesize that MIF might play an essential role in normal bone remodeling; however, over-expressed MIF might have an osteoclastogenic effect on bone metabolism in inflammatory diseases.

We found that MIF-induced RANKL expression in RA synovial fibroblasts was decreased by inhibition of NF-κB, PI3K, STAT3, AP-1, and p38 MAPK, but not ERK and calcineurin. Of the three MAP kinase pathways, only p38 MAPK was involved in MIF-induced RANKL production. In addition, MIF-induced osteoclastogenesis was suppressed by inhibition of NF-κB, PI3K, AP-1, and p38 MAPK, but not by inhibition of JAK/STAT3. These results suggest that there are different signal pathways involved in MIF-induced osteoclastogenesis. Considering that AP-1 is a downstream molecule, MIF seems to induce RANKL production by synovial fibroblasts mainly via NF-κB, PI3K, STAT3, and p38 MAPK, while it promotes OC differentiation from monocyte precursors via NF-κB, PI3K, and p38 MAPK. In recent years, numerous studies have attempted to define the signal transduction pathways of inflammatory cells activated by MIF in RA synovial fluid. MIF promotes cyclooxygenase-2, PGE_2_, and IL-6 expression via p38 MAPK [39]. MIF also upregulates IL-8 and IL-1β via tyrosine kinase-, protein kinase C (PKC)-, AP-1-, and NF-κB-dependent pathways [40]. MIF controls the proliferation of RA synovial fibroblasts, mediated by ERK [36]. The upregulation of MMP-2 by MIF is dependent on PKC, JNK, and Src signal pathways [4]. MIF also upregulates other MMPs including MMP-1 and MMP-3 via tyrosine kinase-, PKC-, and AP-1-dependent pathways [6]. Through the various intracellular signal transduction pathways, MIF activates RA synovial fibroblasts to promote inflammation, cartilage degradation, and bony destruction. In our previous study, we found the induction of MIF is mediated by p38 MAPK pathway when RA synovial fibroblasts are stimulated by conA, IFN-γ, CD40 ligand, IL-15, TGF-β, as well as IL-1β and TNF-α [41]. Among these data, intracellular signal pathways are deeply involved in the pathogenesis of RA. Clinical studies for RA treatment using the inhibitors of different signal pathways such as Syk, p38 MAP, and JAK have been performed until now, and successful results are expected [42-44]. Beyond the inhibition of cytokines or immune cells, oral inhibitors of intracellular molecules will be another choice for refractory RA.

## Conclusions

RA synovial fibroblasts were activated by MIF to produce RANKL, which is mediated by IL-1β, and to promote osteoclastogenesis, which is mediated by RANKL via pathways involving PI3K, p38 MAPK, NF-κB and AP-1. The results add to expand our understanding of the role of MIF in the pathogenesis of bone erosion in human RA, and provide an experimental basis for the development of anti-cytokine agents or target molecules to block intracellular signal pathways in patients who are at high risk of bone destruction or who do not respond to conventional therapy.

## Abbreviations

AP-1: activator protein-1; BM: bone marrow; CTR: calcitonin receptor; DMEM: Dulbecco's modified Eagle's medium; ELISA: enzyme-linked immunosorbent assay; FBS: fetal bovine serum; IL: interleukin; JAK-2: Janus kinase 2; JNK: c-Jun N-terminal kinase; MAPK: mitogen-activated protein kinase; M-CSF: monocyte-colony stimulating factor; MEM: minimal essential medium; MEK1: MAP kinase kinase-1; MIF: macrophage migration inhibitory factor; MMP: matrix metalloproteinases; NF-κB: nuclear factor kappaB; OA: osteoarthritis; OC: osteoclast; ODF: OC differentiation factor; PBMC: peripheral blood mononuclear cell; PBS: phosphate-buffered saline; PGE_2: _prostaglandin E_2_; PI3K: phosphatidylinositol (PI)-3 kinase; PKC: protein kinase C; RA: rheumatoid arthritis; RANKL: receptor activator of nuclear factor kappa-B ligand; rh: recombinant human; RT-PCR: reverse transcription-polymerase chain reaction; SD: standard deviation; SDF: stromal cell-derived factor;STAT3: signal transducer and activator of transcription 3; TBS: Tris-buffered saline; TNF-α: tumour necrosis factor-alpha; TRAP: tartrate resistant acid phosphatase.

## Competing interests

The authors declare that they have no competing interests.

## Authors' contributions

HR Kim and KW Kim designed and performed all experiments and drafted the manuscript. HG Jung, HJ Oh and KS Yoon assisted in designing the study. ML Cho and SH Lee conceived the study and drafted and edited the manuscript. All authors read and approved the final manuscript.
